# Short-term qualitative and quantitative analyses of preseptal injection of hyaluronic acid on the treatment of acquired lower eyelid cicatricial ectropion

**DOI:** 10.5935/0004-2749.2022-0245

**Published:** 2023-03-20

**Authors:** Laryssa K. Veloso, Gabriel Ferreira, Jessica P. Marques, Roberta L.F.S. Meneghim, Alicia F. Galindo, Carlos R. Padovani, Silvana A. Schellini

**Affiliations:** 1 Ophthalmology Department, Medical School, Universidade Estadual Paulista “Júlio de Mesquita Filho”, Botucatu, SP, Brazil; 2 Ophthalmology Department, Rio Hortega University Hospital, Valladolid, Spain; 3 Biostatics Department, Instituto de Biociências, Universidade Estadual Paulista “Júlio de Mesquita Filho”, Botucatu, SP, Brazil

**Keywords:** Ectropion, Cicatrix, Eyelids, Skin abnormalities, Hyaluronic acid, Dermal fillers, Injections, Ectrópio, Cicatriz, Pálpebras, Anormalidades da pele, Ácido hialurônico, Preenchedores dérmicos, Injeções

## Abstract

**Purpose:**

Recently, hyaluronic acid (HA) was proposed as a promising option for the treatment of acquired lower eyelid cicatricial ectropion. However, this effect was not confirmed by quantitative assessments. This study aimed to assess the effect of hyaluronic acid on the treatment of acquired lower eyelid cicatricial ectropion.

**Methods:**

Eight patients with acquired lower eyelid cicatricial ectropion (13 eyelids) were treated with a single 1 mL injection of hyaluronic acid in the preseptal area of the lower eyelid. Evaluation of symptoms and biomicroscopic exam was performed before and 30 days after hyaluronic acid injection. Quantitative analysis of the lower eyelid position (with and without lid traction) was determined before and 30 days after hyaluronic acid injection through standard photographs analyzed using the ImageJ.

**Results:**

All patients experienced partial improvement of symptoms. The lower eyelid position was significantly lifted after hyaluronic acid injection with a significant reduction of medial and lateral angles, reduction of the margin reflex distance, and total and medial ocular fissure area. However, signs of lid margin inflammation and corneal punctate keratitis persisted.

**Conclusions:**

Hyaluronic acid injected in the pre-septal area of the lower eyelid improved acquired lower eyelid cicatricial ectropion symptoms and significantly lifted the position of the lower eyelid. Further studies, with a large number of participants and a long-term follow-up period, are needed to better determine the permanency of the effects of hyaluronic acid injections on the treatment of acquired lower eyelid cicatricial ectropion.

## Abstract (pt)

**Objetivo:**

Recentemente, o ácido hialurônico foi proposto como promissor no tratamento do ectrópio cicatricial adquirido da pálpebra inferior. No entanto, não foram feitas avaliações quantitativas para confirmar este efeito, motivo que levou a realização do presente estudo que visou avaliar o efeito do ácido hialurônico no tratamento do ectrópio cicatricial adquirido da pálpebra inferior.

**Métodos:**

Oito portadores de ectrópio cicatricial adquirido da pálpebra inferior (13 pálpebras) foram tratados com uma única dose de 1 mL de ácido hialurônico, injetada na área pré-septal da pálpebra inferior. Os sintomas e o exame biomicroscópico foram realizados antes e 30 dias após a injeção do ácido hialurônico. A análise quantitativa da posição palpebral inferior (com e sem tração palpebral) foi determinada antes e 30 dias após a injeção do ácido hialurônico por meio de fotografias que foram analisadas usando o programa ImageJ.

**Resultados:**

Todos os pacientes apresentaram melhora parcial dos sintomas. A posição da pálpebra inferior foi elevada significativamente após a injeção do ácido hialurônico, com redução significativa dos ângulos medial e lateral, da distância entre o reflexo pupilar e a margem da pálpebra inferior, da área de fissura palpebral total e da área medial. No entanto, sinais de inflamação da margem palpebral e ceratite puntata da córnea persistiram.

**Conclusões:**

O ácido hialurônico injetado na área pré-septal da pálpebra inferior, melhorou os sintomas do ectrópio cicatricial adquirido da pálpebra inferior e elevou significativamente a posição da pálpebra inferior. Estudos com maior número de participantes e período de acompanhamento mais longo são necessários para melhor determinar os efeitos das injeções de ácido hialurônico a longo prazo no tratamento do ectrópio cicatricial adquirido da pálpebra inferior.

## INTRODUCTION

Hyaluronic acid (HA) is a glycosaminoglycan disaccharide biopolymer composed of alternated or repeated polyanionic units of D-glucuronic and N-acetyl-D-glucosamine. It is a natural component of the human skin, with a low degree of immunogenicity, thus being a safe and effective dermal filler^1^,^2^.

In addition to the esthetic use, HA can be an alternative to skin graft surgery to treat congenital^3^,^4^ or acquired lower eyelid cicatricial ectropion (ALECE)^5^,^6^,^7^,^8^. However, no quantitative assessments in the literature have confirmed the effect of HA injected in the pre-septal area on the treatment of ALECE.

In this study, we aimed to analyze whether HA injected in the pre-septal area of the lower eyelid of patients with ALECE can improve symptoms and significantly lift the lower eyelids.

## METHODS

This prospective interventional study was approved by the Human Research Ethics Committee of the Medical School of the State University of São Paulo (UNESP), São Paulo, Brazil, and all participants signed the informed consent form before the procedures.

The **inclusion criteria** were as follows: primary cicatricial ectropion carriers resulting from sun exposure; secondary carriers resulting from previous eyelid surgery, with no age or sex restriction; and severe, moderate, or mild ectropion that affected the whole lower eyelid or just a part of it.

The **exclusion criteria** were as follows: other types of eyelid ectropion, patients with coagulopathies, or patients using anticoagulant medications.

**HA administration**: A single dose of 1 mL of HA (Restylane Lidocaine®, Q-Med AB, Uppsala, Sweden) was injected using a needle kit (Caliber of 2 × 29 G × 1/2 thin walls, Q-Med AB) in multiple small punctures along the infraciliary pre-septal region up to the orbital border of the affected lower eyelid in the suborbicular plane. To avoid intravascular injections, aspiration was performed before the procedure.

### Evaluated parameters

Symptom and slit-lamp ocular examinations for posterior blepharitis, keratitis, or other biomicroscopic changes in the ocular surface were performed before and 30 days after HA injection.

Quantitative parameters were evaluated using a ruler and standardized digital photos were taken before and 30 days after HA injection. The patient was positioned with the head fixed in the slit-lamp chin in the primary gaze position, and a Nikon Coolpix E5000 (8.4 V/0.9 A, Japan) was positioned at a standardized distance from the patient; flash was used. The images were taken in the same room, with constant lighting and without air conditioning. The standard unit to be considered was obtained by using two conventional rulers, fixed on the top and the side of the slit-lamp chin.

The lower eyelid was photographed either relaxed or with downward traction by the examiner’s index finger up to its maximum traction. The same examiner performed all procedures for all patients.

**Figure 1:**
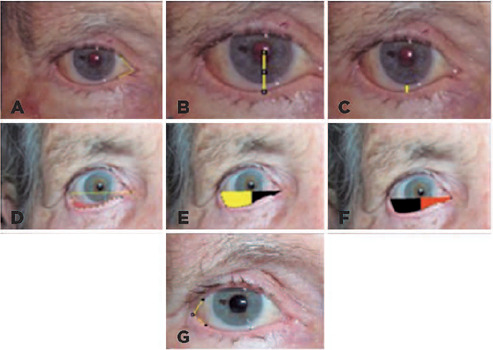
Measurements method using Image J. (A) Internal angle (IA). (B) Inferior margin reflex distance (MRD2). (C) Distance between the lower limbus and the lower palpebral margin (DLL). (D) Total area (TA). (E) Lateral area (LA) (yellow). (F) Medial area (MA) (orange). (G) External angle (EA).

Digital images were then transferred to a computer (Dell Inspiron Intel® Core™ i3 5010U, 2.10 GHz) and analyzed using ImageJ (version 1.55, NIH, USA). The following measurements were performed: medial and lateral lid angles (grades), distance between the corneal reflex and the lower eyelid margin (MRD2) (cm), distance between the lower limbus and the lower eyelid margin (cm), total inferior ocular fissure area (delimited by a line passing through the medial and lateral commissures and another line following the lower eyelid margin, cm^2^), lateral area (lateral portion of the total area divided by a vertical line passing through the pupillary reflex up to the lower eyelid margin; cm^2^), medial area (medial portion of the total area divided by a vertical line passing through the pupillary reflex up to the lower eyelid margin; cm^2^) (Figure 1).

Data were statistically analyzed using Student’s t-test, Mann-Whitney test, and multivariate analysis of variance complemented with the Bonferroni multiple comparison test, and the adopted p-value was 5%.

## RESULTS

The included patients were 74.3 ± 5.4 years old, white-skinned, 87.5% were male, and 80% were farm workers. ALECE was unilateral in 2 (25%) patients and bilateral in 6 (75%).

According to the eyelids, ALECE was caused by primary cicatricial ectropion in 6 (46.1%) eyelids and was secondary to previous blepharoplasty in 6 (46.1%) and tumor removal in 1 (7.7%). ALECE was severe in 20%, moderate in 50%, and mild in 30%. The entire lower lid was affected in 90% of patients, whereas only the lateral portion was affected in the remaining 10%. Laxity was severe, moderate, and mild in 40%, 40%, and 20% of the cases, respectively.

Thirteen eyelids received a single 1-mL dose of HA in multiple small punctures along the infraciliary pre-septal region up to the orbital border of the affected lower eyelid in the suborbicular plane.

The main symptoms of ALECE carriers before the HA injection were epiphora (100%), eye-burning sensation (80%), or ocular discharge (70%). The most frequent ocular signs detected by the anterior segment examination were posterior blepharitis (90%), conjunctival hyperemia (60%), and punctate keratitis (60%).

During the injection, most of the patients complained of mild pain (60%). After the injection, the majority had ecchymosis (80%) or Tyndall effect (40%), with complete remission within 30 days.

Additionally, 30 days after the injection, 40% of the patients had a reduction in epiphora and eye-burning sensation. However, posterior blepharitis, conjunctival hyperemia, and punctate keratitis did not change.

Quantitative measurements, before and 30 days after HA injection, are presented in table 1. After the injection, significant differences were observed even when the lower lid was relaxed, for instance, a reduction of the medial and lateral eyelid angle, a decrease in MRD2, and a reduction in the total inferior ocular fissure area and medial area. Similarly, significant differences were observed when the lower eyelid was at maximum downward traction, for instance, a reduction in the lateral eyelid angle, a decrease in MRD2, a reduction in the distance between the lower limbus and the lower eyelid margin, and a reduction of the total inferior, lateral area, and medial ocular fissure area.

**Table 1:** Mean ± standard deviation of acquired cicatricial eyelid ectropion measurements before and after hyaluronic acid injection with or without downward traction of the lower eyelid

	Without traction			With traction		
	Before injection	After injection	p-value	Before injection	After injection	p-value
Medial angle	62.3 ± 8.8	59.8 ± 8.1	p<0.01	79.6 ±11.3	77.2 ± 9.5	p>0.05
Lateral angle	91.1 ± 14.3	84.5 ± 15.6	p<0.01	107.1 ± 13.4	101.6 ± 12.6	p<0.05
Margin reflex distance 2	1.0 ± 0.2	0.8 ± 0.2	p<0.001	1.7 ± 0.3	1.4 ± 0.3	p<0.001
Lower limbus distance	0.4 ± 0.2	0.3 ± 0.3	p>0.05	1.1 ± 0.3	0.8 ± 0.3	p<0.005
Total area	1.2 ± 0.5	0.9 ± 0.3	p<0.05	2.0 ± 0.6	1.3 ± 0.4	p<0.001
Lateral area	0.6 ± 0.2	0.6 ± 0.3	p>0.05	1.0 ± 0.4	0.7 ± 0.4	p<0.001
Medial area	0.5 ± 0.2	0.3 ± 0.1	p<0.05	1.0 ± 0.3	0.7 ± 0.3	p<0.005

## DISCUSSION

This case series assessed whether HA injection can improve the lower eyelid position of patients with ALECE. We noticed that HA injection in the pre-septal area of the lower eyelid reduced symptoms, whereas quantitative evaluation revealed that the lower eyelid position was significantly lifted, reducing the ocular surface area exposed and the eyelid ectropion.

ALECE is a malposition of the lower eyelid caused by the retraction of the anterior lamellae of the eyelid, which can be primary, caused by intense sun exposure of a predisposed fair skin, or secondary, caused by trauma, injury, or previous surgery. The conventional treatment is based on surgical procedures, depending on the severity and location of the eyelid eversion. Recently, HA injection in the pre-septal area of the lower eyelid has been suggested to treat cicatricial and involutional ectropion, a procedure that is easily performed in the office, is less expensive, has fewer risks than a surgical procedure, and has a short work downtime^7^.

Our patients reported symptom reduction after HA injection, despite persistent signs of blepharitis, hyperemia, and punctate keratitis, probably because of chronic eyelid margin inflammation, which is frequently associated with ALECE.

As previously reported^5^,^9^, we also observed ecchymosis and the Tyndall effect, probably because of HA injection within the superficial plans over the orbicularis muscles, which is very well vascularized, increasing the risk of ecchymosis. In contrast with a previous study in which lumpiness was reported in 63.7% of patients who received an HA injection^6^, none of our patients developed this cosmetically unacceptable result. Other possible complications related to HA fillers such as irregular fullness, bruising, overcorrection, and nodulation^10^were not observed.

Indeed, these events were minimized with a good technique, i.e., by injecting the filler into deeper skin layers. Furthermore, no serious complications such as cellulitis, persistent edema, visual loss, or skin necrosis were observed^11^,^12^. In addition, the risk of severe or vision-threatening complications related to lower eyelid periorbital injections is low 8.

The quantitative evaluation after HA injection confirmed that the lower eyelid was significantly lifted, reducing the ectropion and thus the exposed ocular surface area. Nevertheless, none of our patients had complete remission of ALECE. This partial response may be related to the severity of ALECE, degree of associated ligament laxity, or insufficient volume of HA injected. Better results might be achieved in mild ALECE cases by using more than a single HA injection, increasing the amount of HA injected, or associating the HA injection with surgical procedures to reduce ligament laxity.

As the ligaments weaken, eyelid laxity occurs, and the eyelid can evert to an ectropion or reverse to an entropion, depending on other concomitant factors. Patients with ALECE have short anterior lamellae, with a lower direction vector of the eyelid margin, which when associated with a secondary increase in the horizontal length of the eyelid can accentuate the laxity of eyelid ligaments.

These factors may worsen the eyelid margin eversion, defaulting excreting the secretions of the Meibomian glands, forming a vicious cycle involving posterior blepharitis, keratinization of the tarsal conjunctiva, and punctate keratitis, resulting in the exposure of the conjunctiva to the aggressive environmental factors.

With the downward traction of the lower eyelid to its maximum, the effect of horizontal eyelid laxity on the outcomes of HA injection could be evaluated. The lower eyelid is a free arch, with strong ligament adhesion on its inner and outer corners. In addition, the medial tendon is stronger than the lateral^13^. Our measurements with traction corroborated this finding because the medial angle and medial area values varied less than the lateral angle and lateral area values.

Partial or total improvement of ALECE was qualitatively reported^6^. In this study, we registered a partial improvement of the lower eyelid position after HA injection in ALECE carriers both qualitatively (symptom reduction) and quantitatively. The lifting of the lower eyelid by HA injection is probably due to the expansion of the retracted anterior lamella, elevating the eyelid margin. Moreover, mechanical stress caused by HA can stretch fibroblasts, consequently stimulating collagen production. This behavior was demonstrated in vivo and in vitro^14^.

The improvement of the lower eyelid position is impaired with time, with gradual recurrence of lid retraction. Therefore, a limitation of this short-term study is the observation period (30 days after the procedure) and the small sample size. A long-term follow-up and a large number of participants can clarify whether the eyelid position remains unchanged over time, as well as the required frequency of injections and amount of HA needed for a good eyelid positioning. Current evidence reveals residual effects, even 6 months after HA injection^8^, and qualitative evidence shows good correction for periods longer than 1 year^5^, or even in 1.5 years^7^.

In conclusion, to the best of our knowledge, this is the first study that quantitatively assessed changes in the lower eyelid position using HA injection in patients with ALECE. Short-term qualitative and quantitative data showed that HA had a positive effect, i.e., elevating the lower eyelid position. Further studies with a large number of patients and a long-term follow-up are needed to determine more precisely the efficacy of HA injections in the treatment of ALECE.
